# Molecular Typing of Ukrainian *Bacillus anthracis* Strains by Combining Whole-Genome Sequencing Techniques

**DOI:** 10.3390/microorganisms10020461

**Published:** 2022-02-17

**Authors:** Hanka Brangsch, Anatolii Golovko, Nataliia Pinchuk, Oleg Deriabin, Tetiana Kyselova, Jörg Linde, Falk Melzer, Mandy Carolina Elschner

**Affiliations:** 1 Institute for Bacterial Infections and Zoonoses, Friedrich-Loeffler-Institut, Naumburger Str. 96a, 07743 Jena, Germany; joerg.linde@fli.de (J.L.); falk.melzer@fli.de (F.M.); mandy.elschner@fli.de (M.C.E.); 2 Department of Bacteriological Research and Quality Control of Veterinary Immunobiological Preparations, SSCIBSM, 30, Donetskaya St., 03151 Kyiv, Ukraine; anatolii.golovko@gmail.com (A.G.); pinchuk.2578@gmail.com (N.P.); don.lmb@gmail.com (O.D.); korba2301@gmail.com (T.K.)

**Keywords:** anthrax, *Bacillus anthracis*, genome-sequencing, ONT, genotyping

## Abstract

Anthrax is a recurrent zoonosis in the Ukraine with outbreaks occurring repeatedly in certain areas. For determining whether several *Bacillus anthracis* genotypes are circulating in this region, four strains from various sources isolated from different regions of the Ukraine were investigated. By combining long- and short-read next-generation sequencing techniques, highly accurate genomes were reconstructed, enabling detailed in silico genotyping. Thus, the strains could be assigned to the Tsiankovskii subgroup of the “TransEurAsia” clade, which is commonly found in this region. Their high genetic similarity suggests that the four strains are members of the endemic population whose progenitor was once introduced in the Ukraine and bordering regions. This study provides information on *B. anthracis* strains from a region where there is little knowledge of the local population, thereby adding to the picture of global *B. anthracis* genotype distribution. We also emphasize the importance of surveillance and prevention methods regarding anthrax outbreaks, as other studies predicted a higher number of cases in the future due to global warming.

## 1. Introduction

The importance of reliable infection surveillance and tracing of infection chains, whether of bacterial or viral origin, has become most evident in light of the SARS-CoV2 pandemic [1], but has already been recognized before for other microorganisms. The bacterial agent of anthrax, *Bacillus anthracis*, is one of the pathogens whose occurrence is notifiable to the responsible authorities, like the European Centre for Disease Control and Prevention for cases in European countries.

Anthrax is a zoonotic disease mainly affecting ruminants at pasture, while most human cases are attributed to handling or consumption of contaminated animal products [2,3,4]. It is of public concern, not just because of its potential in biological warfare, but also as a re-occurring disease in certain parts of the world with 20,000–100,000 reported anthrax cases worldwide per year [5]. Although anthrax outbreaks occurred frequently in Europe in the past, they now are rare due to strict measures, e.g., burning of carcasses, vaccination programs and the ban of bone meal imports [6,7,8,9].

However, *B. anthracis* is endemic in some regions of Europe, e.g., Italy, where sporadic cases and outbreaks are reported every year, mainly during the summer months [10]. Sources of outbreaks can be old carcass burial sites or human activities that interfere with the soil surfaces leading to spore exposure [9,11,12]. The problem of lacking knowledge and records on anthrax-diseased animal burial sites has been reported from several countries [7,13], which is problematic, as *B. anthracis* survives for decades as dormant spore in soil [2] and from there it can be spread by livestock and free-roaming wildlife [14,15].

Besides outbreaks caused by endemic strains, recent anthrax cases in Europe can be ascribed to strains imported from other continents [3,6], the most prominent incident being human anthrax cases caused by contaminated heroin in several European countries in 2000 to 2013. By analysing the genome sequences, it was proven that outbreaks reported from Denmark, Norway, France, Germany and the United Kingdom between 2000 and 2013 were connected and the responsible strains came from two separate contamination events, but originated from the same geographic region, possibly the Middle East or Turkey [16,17]. Thus, whole-genome sequencing enables follow-up on infection chains and reveals deeper phylogenetic relations.

As a consequence of its life cycle, *B. anthracis* evolution is mainly restricted to growth phases inside the host (20–40 generations). Thus, time for mutations or horizontal gene transfer is confined to a short time span and strains exhibit highly monomorphic genomes that complicate strain differentiation, posing a challenge for classification techniques [18,19]. As high-throughput genome sequencing has become affordable in the last decade, the classical PCR-based typing approaches are being replaced by in silico analysis. New methods have been developed, like the detection of genome-wide single-nucleotide polymorphisms (SNPs) and core genome multilocus sequence typing (cgMLST), where, in the case of *B. anthracis*, 3803 genes are analysed simultaneously [20]. Both methods allow for higher phylogenetic resolution than the PCR-based analysis of a set of 12 to 19 canonical SNPs, which is often used for classification of *B. anthracis* [16,21,22].

Globally, the *B. anthracis* population can be divided into three major clades (A, B and C) sharing canonical SNPs [22]. The majority of *B. anthracis* strains investigated so far belong to the “TransEurAsia” (TEA) clade, a sub-group of the A clade, which is distributed across a large geographic area, and some sub-clades are dominated by strains from Eastern Europe [20,23,24]. The geographic origin of *B. anthracis* is still under debate, but it is assumed that trade routes and movement of armies benefitted the spread of anthrax throughout the world [9,22].

The Ukraine is located in Eastern Europe and is a former Soviet Union member state whose economy is mainly based on agriculture. As in other neighbouring countries, anthrax frequently occurred at the beginning of the last century with several reported hotspots distributed all over the country. Over 23,400 outbreaks in 8927 sites have been reported between 1913 and 2012. Although cases have decreased since 1979, multiple anthrax foci are still assumed [8]. In a modelling study, Carlson et al. [5] predicted a comparably high probability of anthrax occurrence in this region. That the disease is still of importance in the Ukraine is substantiated by recent reports of outbreaks among wild-living boars, livestock and a human case due to contact with an infected pig [8,15,25,26].

However, little publicly accessible information is available on whole-genome sequencing (WGS) data of strains isolated on Ukrainian territory and in bordering countries, i.e., no genome sequences of strains isolated in the Ukraine are publicly available. In this study, we wanted to close this gap of knowledge by analysing four strains that were isolated from various sources at different locations across the Ukraine. We are well aware that three of the strains have been characterized by PCR before [25]. However, by applying Illumina short-read in combination with Oxford Nanopore (ONT) long-read technology, we obtained the currently most detailed picture of the genotype affiliation, which supports the accurate classification of these strains from an underrepresented geographic region, and thereby enables tracing infection chains in case of outbreak events.

## 2. Materials and Methods

### 2.1. Strain Origin

**Figure 1:**
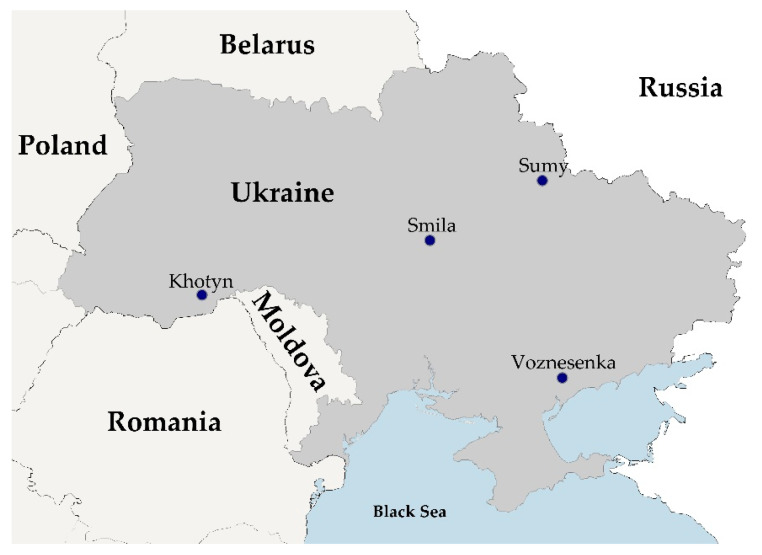
Map of the Ukraine with location of sampling sites, generated with Ridom SeqSphere+ v7.7.

Four potential *B. anthracis* strains that have been isolated in four different Ukrainian oblasts (provinces) in 2010 and 2012 from animals and soil (Figure 1, Table 1) were obtained from the strain collection of the State Scientific Control Institute of Biotechnology and Strains of Microorganisms (SSCIBSM). BA-D12-MEL was isolated in Southern Ukraine from a domestic dog that was fed meat from an infected heifer, the first documented case of anthrax in a dog in this country [26]. For the other three strains, no published reports were available.

### 2.2. DNA Isolation and Sequencing

Strains were grown on nutrient agar (Merck, Darmstadt, Germany) in petri dishes for 24 h at 37 °C, and the biomass was harvested and used for subsequent DNA isolation. For short-read sequencing, DNA was isolated using the Genomic-tip 100/G kit (Qiagen, Hilden, Germany) according to the manufacturer’s recommendations. For long-read sequencing, DNA was extracted using the NucleoBond HMW DNA kit (Macherey-Nagel, Düren, Germany). The genomic DNA content and purity were checked by Qubit 3.0 Fluorometer (QubitTM DNA HS Assay, Life Technologies, Thermo Fisher Scientific Inc., Eugene, OR, USA) and Colibri Microvolume Spectrometer (LB 915, Berthold Technologies GmbH & Co. KG, Bad Wildbad, Germany) measurement. Sequencing library preparation was performed using the Nextera XT library preparation kit (Illumina Inc., San Diego, CA, USA) for Illumina sequencing and the Ligation Sequencing Kit SQK-LSK 109 in combination with the Barcoding Kit EXP-NBD 104 (Oxford Nanopore Technologies Ltd., Oxford, England) for ONT, both according to the manufacturer’s recommendations. The Illumina libraries were sequenced on a MiSeq system (Illumina) producing paired-end reads. For long-read sequencing, the libraries were run on a MinION Mk1B sequencing device (Oxford Nanopore Technologies Ltd.) for 24 h using a R9.4.1 flow cell.

### 2.3. Processing of Sequencing Data

Data generated by MiSeq sequencing was analysed with a Linux-based in-house pipeline, WGSBAC v2.2 (https://gitlab.com/FLI_Bioinfo/WGSBAC/-/tree/version2). In this framework, sequencing quality was checked and reads were assembled by Shovill v1.0.4 (https://github.com/tseemann/shovill). The identity of the isolates as *B. anthracis* was verified by kraken2 [27], which also checks for contaminations. Annotation of coding sequences and RNA-coding genes was done using Prokka v1.14.5 [28].

For incorporation of ONT data, the raw sequencing results were subjected to basecalling applying the “super-accuracy” model of Guppy basecaller v5.0.7 (Oxford Nanopore Technologies Ltd.), demultiplexing and assembly including polishing with the corresponding MiSeq read data using microPIPE [29]. Sequencing quality was controlled using NanoPlot v1.32.1 [30]. Statistics for all assemblies were obtained using Quast v5.0.2 [31]. *B. anthracis* virulence plasmids pX01 and pX02 were detected by in silico PCR (perl script “in_silico_pcr”; https://github.com/egonozer/in_silico_pcr) with primers pX01-for (CAATTTATTAACGATCAGATTAAGTTCA)/pX01-rev (TCTAGAATTAGTTGCTTCATAATGGC) and pX02-for (TCATCCTCTTTTAAGTCTTGGGT)/pX02-rev (GTGTGATGAACTCCGACGACA) [32]. Raw sequencing data and assemblies were submitted to the European Nucleotide Archive (ENA) under the project number PRJEB49261.

### 2.4. Comparison to Public Database Entries

In order to compare the strains to the global *B. anthracis* population, entries available in October 2021 in the public repositories Sequence Read Archive (SRA) and NCBI GenBank were selected and processed bioinformatically as described before. The entries were selected based on their affiliation to different canSNP groups, as given in Sahl et al. [23], and geographic origin in the wider vicinity of the Ukraine. To ensure the identity of these strains as *B. anthracis*, the average nucleotide identity (ANI) to the reference genome, *B. anthracis* ‘Ames Ancestor’ (GCF_000008445.1) was calculated using fastANI v1.1 [33] and a threshold of at least 99.9% nucleotide identity for further processing (Supplementary Table S1). In total, 80 foreign strains were selected, different subsets of which were used in the genotype analysis (Supplementary Table S2).

### 2.5. Genotyping

Multilocus Variable Number of Tandem Repeats (MLVA-31) genotyping and canonical SNP calling was performed in silico with the tools MISTReSS (https://github.com/Papos92/MISTReSS) and CanSNPer v1.0.8 [34], respectively, as included in the WGSBAC pipeline. MLVA profiles served as input for construction of a minimum spanning tree by GrapeTree v1.0 [35]. Genomic distance based on core genome SNPs was determined using either Parsnp v1.2 within the Harvest suite [36] or snippy (https://github.com/tseemann/snippy) in conjunction with RAxML v8.2.12 [37] for phylogenetic tree construction. In all cases, *B. anthracis* strain ‘Ames Ancestor’ (GCF_000008445.1) served as the reference genome. Furthermore, genotyping was performed by means of core genome multilocus sequence typing (cgMLST) using Ridom SeqSphere+ v7.7 [38] with the scheme of Abdel-Glil et al. [20]. For tree visualisation, FigTree v1.4.3 (http://tree.bio.ed.ac.uk/software/figtree/) was used and publication-ready figures were created with Inkscape v1.1 (https://inkscape.org).

## 3. Results

### 3.1. Genome Sequencing

Illumina as well as ONT sequencing yielded enough data at a coverage of at least 85-fold, allowing for genome assembly. However, the quality of the assemblies differed. Assemblies generated from Illumina short-read sequencing data were smaller and more fragmented, covering a lower fraction of the reference genome (Table 2). From ONT data, all three expected contigs (chromosome and two plasmids) could be reconstructed. However, compared to the reference genome, the genomes exhibited almost double the number of insertions and deletions (indels) than those generated by Illumina reads. The polishing of the ONT-based genomes with Illumina reads markedly reduced indel frequency. Thus, these combinatorial genomes were selected for further analysis.

All genomes exhibited a GC content of 35.25%. In silico PCR detected plasmid markers in all four strains, substantiating the correctness of the assembly of three contigs per strain. No other plasmids were detected. Genome sizes as well as number of predicted coding sequences and RNA-coding genes matched those of the ‘Ames Ancestor’ reference genome (see also Supplementary Table S3).

### 3.2. Genotyping

**Figure 2:**
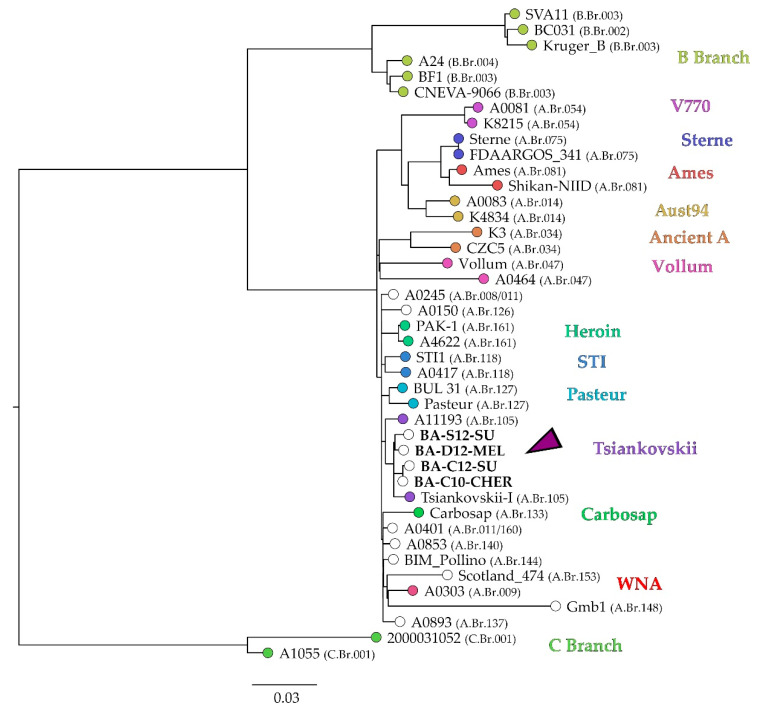
Approximately maximum-likelihood tree based on cgSNP differences of global *B. anthracis* strains, generated with Parsnp with affiliation to CanSNP groups according to Sahl et al. [23]. As reference genome, *B. anthracis* ‘Ames Ancestor’ (GCF_000008445.1) was used. Coloured tips indicate the genetic group nickname, while for empty circles no genetic group name has yet been assigned. The arrow indicates the position of the Ukrainian strains. Values in brackets indicate CanSNP group (see also Supplementary Table S2) and the scale bar indicates the number of nucleotide changes per site.

Canonical SNP calling identified the strains from the Ukraine as members of the A.Br.008 clade. However, as recently this clade was further subdivided in different groups [23,24], cgSNP analysis was performed for a more detailed placement of the four Ukrainian strains within the global *B. anthracis* phylogeny. Their genome sequences were mapped to the ‘Ames Ancestor’ reference genome together with 37 isolates representing the major genetic groups. In total, 5312 core genome SNPs were identified and used for construction of an approximately maximum-likelihood tree (Figure 2).

The strains from the Ukraine fell into the A clade of the global phylogeny, more precisely, the TransEurAsian subclade that comprises, among others, the groups ‘Heroin’, ‘STI’, ‘Pasteur’, ‘Tsiankovskii’, ‘Carbosap’ and ‘WNA’.

The investigated strains all clustered together with strains of the so-called Tsiankovskii group (A.Br.105), whose depicted representatives were isolated in Russia (Tsiankovskii-I) and Bulgaria (A11193) (Figure 2). Between 44 and 62 SNPs discriminated the strains from the Ukraine from each other, except for BA-C10-CHER and BA-C12-SU, which were separated by 16 SNPs (Supplementary Table S4). The similarity between strains from the Ukraine was higher than to the Russian vaccine strain Tsiankovskii-I.

**Figure 3:**
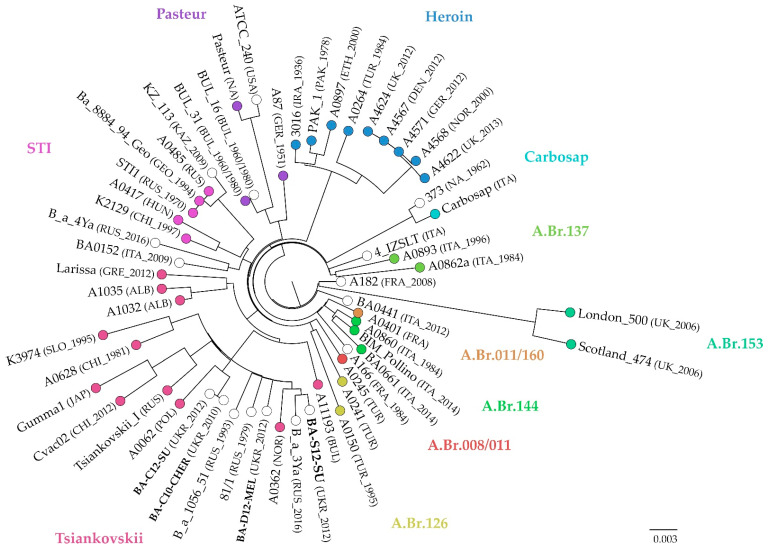
Neighbour joining tree based on cgMLST including strains of the TEA group. Colours indicate affiliation to CanSNP groups according to Sahl et al. [23]. Information in brackets state country and year of isolation, if known. Ukrainian strains are given in bold letters. The scale bar indicates substitutions per site.

Compared to canSNP analysis, a more extensive typing of the strains was achieved by applying cgMLST to TAE group members, where alleles of 3808 genes were assessed. Here, 53 strains from the TEA clade were included in the analysis. On average, 99.3% of targeted alleles were called in each strain, spanning from 92.8% to 100% identified variants (Supplementary Table S2). The resulting neighbour joining tree (Figure 3) confirmed the affiliation of the Ukrainian strains to the Tsiankovskii group. The Ukrainian strains differed in 25 to 29 alleles from each other, again with the exception of BA-C10-CHER and BA-C12-SU, between which merely one allele differed. When comparing the isolates to foreign strains, the closest match was between BA-S12-SU from Sumy in the northeast of the Ukraine and a Russian isolate, B-a-3Ya, from soil in the East Siberian Lowland with 17 alleles difference. Allelic differences to other Russian strains, i.e., Tsiankovskii-I or 81/1 and B-a-1056_51 from Stavropol, were comparable to those among the majority of the Ukrainian strains (27–35 allele difference). However, cgMLST also showed allele profile concordance with strains from Eastern Europe, i.e., Bulgaria, Poland, Slovakia and Albania. In the neighbour joining tree, the similarity to strains from Russia was most pronounced for BA-D12-MEL and BA-S12-SU, isolated in eastern and southern Ukraine, while the other two strains clustered with a Polish isolate. A0628, a soil isolate from China, might be a derivative of the Russian vaccine strain Tsiankovskii-I, like Cvac02, which would explain its appearance in this cluster.

**Figure 4:**
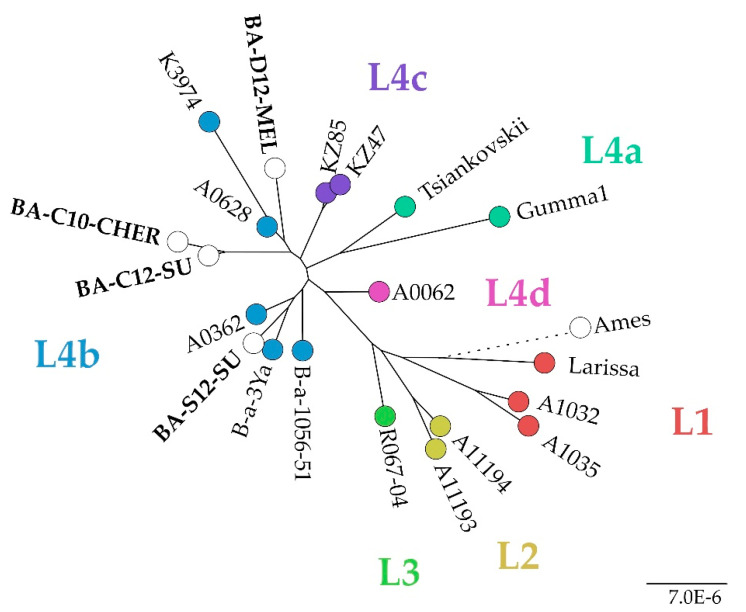
Maximum Likelihood tree based on SNP typing of strains of the Tsiankovskii clade, generated with snippy. Colours indicate affiliation to sublineages within the group, according to Shevtsov et al. [39]. The scale bar indicates the number of nucleotide changes per site.

In a cgSNP analysis exclusively including members of the Tsiankovskii group (Figure 4), it was found that the Ukrainian strains belonged to the sublineage L4, which was recently established and is currently dominated by strains from Russia, Eastern Europe and Kazakhstan [39]. 730 SNPs were called in this analysis. The strain BA-S12-SU, isolated in Sumy, which is located in the East of the Ukraine, clustered differently within this sublineage than the other three investigated strains.

**Figure 5:**
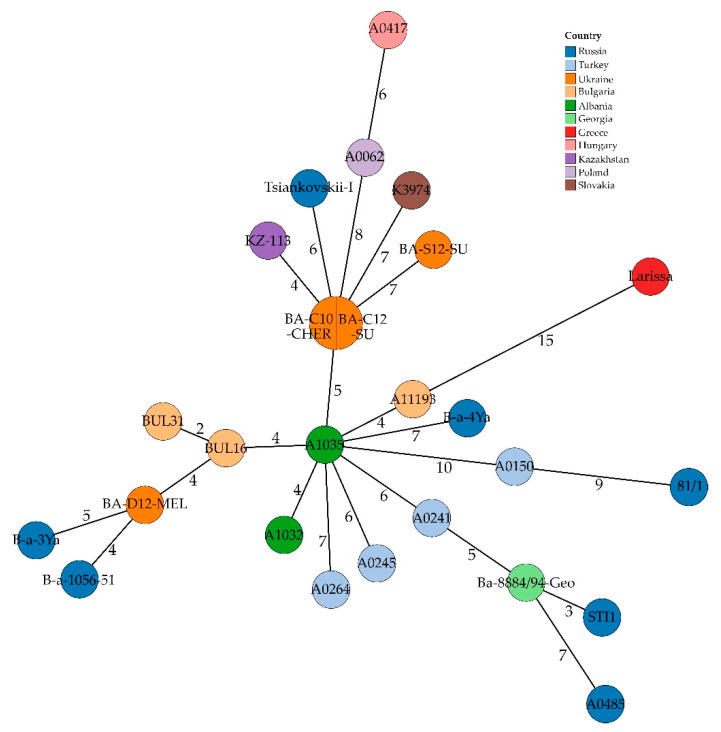
Minimum Spanning Tree based on in silico MLVA data, i.e., number of different alleles, of Ukrainian *B. anthracis* strains and strains from wider neighbouring regions. Numbers indicate allelic differences between samples.

Regarding MLVA data, the in silico approach of the present study confirmed the results of Biloivan et al. [25] regarding the identical MLVA profiles of BA-C10-CHER and BA-C12-SU (Figure 5, Supplementary Table S5). The divergence between other investigated strains was five loci. This analysis benefitted from the combinational approach of polishing ONT assemblies with Illumina short-reads, as in most Illumina-only assemblies not for all loci an allele could be assigned. In the case of BA-S12-SU, half of the loci were missing, while in the polished assembly the profile was complete (Supplementary Table S5).

In accordance with cgMLST results, strains from Russia and Eastern Europe showed the highest allelic profile similarity. Additionally, an isolate from Kazakhstan (KZ-113) clustered with three of the Ukrainian strains.

Overall, the typing approaches confirmed that the Ukrainian strains exhibited a high level of genomic congruence and showed high similarity to other strains from Russia and Eastern Europe. Especially, the isolates from Central (BA-C12-SU) and Eastern Ukraine near the Romanian border (BA-C10-CHER) were highly congruent in SNPs and allelic profiles, although they were collected two years apart.

## 4. Discussion

There is little to no publicly accessible information on genome sequences of strains isolated in the Ukrainian territory and neighbouring countries. In the present study, we aimed at closing this gap of knowledge by sequencing the genomes of four strains from different regions of the Ukraine and placing them in the frame of the global *B. anthracis* population. By combining two sequencing methods, Illumina and Oxford Nanopore technologies, we could fully reconstruct the genomes of these strains, thereby enabling in-depth typing at a high resolution. In this approach, the high error rate of the long ONT reads is compensated for by polishing ONT assemblies with more accurate Illumina reads [29]. Thereby, also both *B. anthracis* virulence plasmids were correctly assembled in all strains. The in silico MLVA results are in accordance with those generated by PCR [25], underling the correctness of the sequencing and assembly approach. In three of the four strains, complete MLVA profiles could not be generated when using Illumina-only assemblies, rendering this approach unsuitable for in silico MLVA. Thus, for epidemiological studies, the combination of long- and short-read sequencing should be preferred.

There have been efforts for the implementation of routine WGS in the surveillance of bacterial pathogens in the public health sector, e.g., *Staphylococcus aureus*, *Salmonella enterica* [40,41,42]. For this purpose, the portability of the MinION device is advantageous, as it can be used for rapid on-site analysis with limited equipment [42]. The availability of rapid library preparation protocols enables a quick response to outbreak events: genome sequencing of anthrax can be accomplished in less than nine hours, including library preparation [43]. However, subsequent sequencing with short-read technologies is required for a high-genome resolution, in order to detect (additional) plasmids and potential genetic manipulation [44,45,46].

The SNP and cgMLST trees in this study based on the sequencing results and international data are in accordance with clade differentiation of the TAE group observed by other authors [24,47]. Although, the allele-based cgMLST method does not allow for phylogenetic reconstructions, but merely indicates the extent of genomic similarity between strains for enabling epidemiological conclusions. It could be shown that allele- as well as SNP-based approaches yielded largely congruent clustering, as observed in other studies [20,48]. The TEA polytomy derived from cgSNP analysis did not enable the robust differentiation between sublineages of this clade that was observed by Shevtsov et al. [39], despite the usage of the same reference genome for SNP calling. Differences may have arisen from the usage of a core genome SNP calling approach rather than the whole genome SNP analysis, the utilization of different SNP calling pipelines [49] and processing of raw data. The incongruence in tree topology might be caused by the different tree construction methods (maximum likelihood vs. maximum parsimony), whose influence on tree topology has been observed before [50].

In an extensive study of isolates from Russia and neighbouring countries, Eremenko et al. [51] found that most strains in these parts can be assigned to the TransEurAsian clade, which is also the globally most abundant clade with wide geographic distribution [20]. The four Ukrainian strains are genetically comparably homogenous, as all belong to its A.Br.105 subgroup, further substantiating the dominance of this clade and especially A.Br.105 in Eastern Europe and Russia [9,20,22,24,51].

The Tsiankovskii group can further be subdivided into four main sublineages [39], which are dominated by strains from Russia and Eastern Europe. The Ukrainian strains clustered with isolates that were also identified in cgMLST to be similar to the former, e.g., isolates from Russia, Norway and Slovenia. However, the SNP-based analysis revealed a larger distance of the Eastern Ukrainian strain (BA-S12-SU) to the others, indicating that diverse branches of the L4 sublineage might circulate in the Ukraine.

However, the *B. anthracis* population in Poland, a neighbour to the Ukraine, was found to be more diverse [52] and also for the Ukraine members of other canSNP groups have been isolated, e.g., in 1957 from a human anthrax case [51]. The latter case resulted from contact with contaminated goatskin from Ethiopia, which explained the affiliation of the isolate to the A.Br.034 (‘Ancient A’) canSNP group. The affiliation of the here-studied strains to a single canSNP group and their low divergence in SNPs and cgMLST alleles does not hint at separate introductory events. Whether the differentiation of Ukrainian strains within the L4 sublineage resulted from different origins or a differentiation from a single source remains elusive. Regardless, it can be assumed that these are representatives of the prevalent endemic *B. anthracis* population. This is in agreement with MLVA-based studies of Ukrainian *B. anthracis* strains [25] that identified two populations, one of which lacked the pX02 plasmid, which was attributed to isolate relatedness to the vaccine strain STI. As all of the presented strains harbour both plasmids, a re-isolation of vaccine strains can be excluded.

Discrepancies between cgMLST and in silico MLVA results regarding the placement of Ukrainian strains in relation to other isolates from similar geographic origin are most likely the result of homoplasy caused by the higher mutation rate of repeat regions [18,53]. Therefore, comparisons to strains from different geographic regions should be interpreted with caution. Nevertheless, the identical MLVA profiles of BA-C10-CHER and BA-C12-SU were in agreement with a high similarity of the two strains in SNP and cgMLST analysis. The difference of four alleles in cgMLST would impute both strains to the same outbreak, as five alleles were found to be the threshold for strains from the same outbreak event [20]. This is remarkable, as the strains were isolated from different outbreaks over 270 km and two years apart, both from cattle. Since 1920, almost 25,000 anthrax outbreaks were reported in the Ukraine and hotspots are distributed all over the country. Although the number of cases has been decreasing since 1979, multiple persistent and newly arising anthrax foci can be assumed [8,25]. Anthrax outbreaks regularly are caused by disturbance of historical burial sites that harbour infected carcasses and serve as an environmental reservoir [8,13,54]. Estimates assume that there are 13,500 such sites in the Ukraine, most of which are neither signposted nor fenced [13]. In the Ukraine, Chernozem and Kastanozem are the prevailing soil types, which are characterized by a high humus content, thus providing ideal conditions for the long-term persistence of anthrax spores in soil [14,55]. Due to the unhindered access to these foci, anthrax could be transmitted by wildlife. Serological tests of wild boars in the Ukraine revealed several hotspots [15]. Two hot spots are located in oblasts Cherkasy and Chernivtsi, while another is at the southern border to Sumy oblast. These are directly in or near places of origin of three herein investigated strains. Anthrax outbreaks among wild boars near the Romanian border already occurred in the 1990s [8]. Wildlife serving as a vector for anthrax might explain the high similarity between BA-C10-CHER and BA-C12-SU from Chernivtsi and Cherkasy, respectively. The anthrax case in a dog (host to BA-D12-MEL) emphasizes the importance of raising public awareness of the danger of *B. anthracis* transmission via contaminated animal products.

## 5. Conclusions

The present study provides the first detailed picture of the Ukrainian endemic *B. anthracis* population based on whole-genome sequencing, adding to the dominance of isolates from Eastern Europe within the A.Br.105 group of the TEA clade. The distant isolation points of the investigated strains across the Ukraine substantiate the prediction of other authors [4] that this country has a high probability for anthrax occurrence. Thus, we emphasize the importance of surveillance and prevention methods, especially with regard to climate change, which might promote anthrax outbreak and dispersal in the future [8]. Regarding strain typing, such a surveillance system would benefit from a combinatorial approach using the two leading long- and short-read sequencing techniques over a single approach, as this might improve identification of infection sources.

## Figures and Tables

**Table 1:** Origin of investigated Ukrainian *B. anthracis* strains.

Strain	Source	Year	Oblast/Province	Rajon/District	City
BA-C10-CHER	cattle	2010	Chernivtsi	Dniester	Khotyn
BA-D12-MEL	dog	2012	Zaporizhia	Melitopol	Voznesenka
BA-C12-SU	cattle	2012	Cherkasy	Cherkasy	Smila
BA-S12-SU	soil	2012	Sumy	Sumy	Sumy

**Table 2:** Results of genome sequencing and assembly of MiSeq short-read (SRS) and Oxford Nanopore (ONT) data separately or in combination by polishing ONT results with Illumina reads (ONT/SRS).

Strain	Method	Coverage	Bases	Contigs	Indels per 100 kbp	N50	L50	RGF% *
BA-C10-CHER	SRS	158	5,461,659	72	4.14	204,605	7	99.095
	ONT	293	5,506,898	3	9.28	5,230,398	1	99.995
	ONT/SRS	-	5,506,660	3	4.42	5,230,171	1	99.995
BA-D12-MEL	SRS	123	5,464,102	77	4.04	172,867	10	99.059
	ONT	356	5,506,420	3	9.07	5,229,931	1	99.995
	ONT/SRS	-	5,506,276	3	4.32	5,229,793	1	99.995
BA-C12-SU	SRS	85	5,450,437	144	4.21	117,282	14	98.762
	ONT	377	5,506,896	3	9.18	5,230,396	1	99.995
	ONT/SRS	-	5,506,662	3	4.51	5,230,173	1	99.995
BA-S12-SU	SRS	160	5,613,511	958	3.98	289,064	7	99.052
	ONT	369	5,506,632	3	8.67	5,230,160	1	99.992
	ONT/SRS	-	5,506,423	3	4.45	5,229,957	1	99.992

* Reference Genome Fraction in %.

## Data Availability

The data presented in this study are openly available in ENA BioProject PRJEB49261 and in the Supplementary Materials.

## Supplementary Materials

The following supporting information can be downloaded at: https://www.mdpi.com/article/10.3390/microorganisms10020461/s1, Table S1: Average nucleotide identity (ANI) of foreign strains to *B. anthracis* Ames Ancestor, Table S2: Metadata of foreign *B. anthracis* strains used in this study, Table S3: Gene annotations predicted in Ukrainian strain genomes, Table S4: cgSNP distance table of Ukrainian and global *B. anthracis* strains based on the reference *B. anthracis* Ames Ancestor, Table S5: MLVA profiles of Ukrainian strains and strains from wider neighbouring regions, determined in silico.
